# TexTNet: a texture-guided hierarchical architecture for prostate cancer Gleason segmentation in whole slide images

**DOI:** 10.3389/fmed.2026.1815031

**Published:** 2026-10-19

**Authors:** Muhammad Arshad, Wang Chengliang, Muhammad Wajeeh Us Sima, Jamshed Ali Shaikh, Salem Alkhalaf, Fahad Alturise

**Affiliations:** 1 Department of Computer Science and Technology, Chongqing University, Chongqing, China; 2 School of Medicine, Ningbo University, Ningbo, China; 3 School of Computer Science and Engineering, South China University of Technology, Guangzhou, Guangdong, China; 4 Department of Information Technology, College of Computer, Qassim University, Buraydah, Saudi Arabia; 5 Department of Information Technology, College of Computer, Qassim University, Buraydah, Saudi Arabia

**Keywords:** deep learning, Gleason pattern, pathology, prostate cancer segmentation, Swin Transformer, texture-guided attention

## Abstract

Prostate cancer is one of the most common malignancies among men, and accurate segmentation of Gleason grades is crucial for effective diagnosis and treatment planning. Current deep learning methods often struggle with fusing complementary features from CNNs and Transformers, primarily relying on naive feature fusion techniques such as concatenation or summation. These methods fail to leverage the complementary strengths of texture and global context, limiting segmentation performance. To address this challenge, we propose TexTNet, a novel texture-guided hierarchical architecture that integrates Local Binary Pattern (LBP) features into the query projection of the Swin Transformer, creating a Texture-Guided Attention mechanism. This approach directs the Transformer’s attention to texture-rich regions such as glandular borders and nuclear clusters, improving segmentation accuracy. We further enhance feature fusion with the Dual Path Squeeze-Fusion-Excitation (DP-SFE) module, which mitigates boundary blurring, and introduce Asymmetric Convolution Blocks (ACB) for efficient feature extraction. Additionally, the Cross-Directional Feature Aggregator (CDFA) ensures precise positional reconstruction and noise suppression during decoder fusion. We evaluate TexTNet on the SICAPv2, PANDA, ProstateX, and HuBMAP datasets, demonstrating superior performance over state-of-the-art methods in prostate cancer segmentation. TexTNet shows promise as a clinical decision-support tool, demonstrating robust performance on complex, real-world histopathological images and the potential to improve diagnostic reliability for prostate cancer grading.

## 1 Introduction

Prostate cancer (PCa) remains the second most frequently diagnosed malignancy among men worldwide and a leading cause of cancer-associated mortality (1). The clinical management of PCa relies heavily on the Gleason Grading System, which stratifies tumors based on the histological architecture of glandular tissue. Pathologists assign a Gleason score by identifying patterns ranging from distinct, well-formed glands (Grade 3) to fused, cribriform, or anaplastic structures (Grades 4 and 5) (2). This grading is the most potent predictor of prognosis and determines the necessity for interventions such as radical prostatectomy or radiation therapy. However, the manual assessment of Whole Slide Images (WSIs) is inherently subjective, labor-intensive, and prone to significant inter and intra observer variability, with disagreement rates among general pathologists reported as high as 30%–50% (3). The critical distinction between Grade 3 and Grade 4 patterns often defined by subtle disruptions in glandular boundaries is particularly challenging, necessitating the development of objective, quantitative, and automated decision support systems (4).

The digitization of histopathology has catalyzed the adoption of Deep Learning (DL) for automated Gleason grading. Early approaches relied on handcrafted features, such as Local Binary Patterns (LBP) (5) and Haralick textures, to capture the chromatin density and stromal characteristics that define tumor grade (6). While these descriptors offered rotation invariant texture analysis, they lacked the capacity to model high-level semantic context. The advent of Convolutional Neural Networks (CNNs), particularly U-Net and its variants, revolutionized the field by enabling end-to-end feature learning. CNNs excel at extracting local hierarchical features; however, their performance is fundamentally limited by the locality of convolutional operations (7). The receptive field of a standard CNN is often insufficient to capture the long-range dependencies required to distinguish between a cluster of tightly packed individual glands (Grade 3) and a large, continuous fused gland (Grade 4). Consequently, CNN-based models frequently struggle with global structural coherence, leading to fragmented segmentation maps (8).

To address the limitations of local convolution, the computer vision community has increasingly pivoted toward Vision Transformers (ViTs) (9). By leveraging self-attention mechanisms, Transformers can model global dependencies across the entire input field, effectively capturing the semantic context required for complex histological grading (10). Hierarchical architectures, such as the Swin, have further optimized this by introducing shifted windows to maintain computational feasibility while preserving local and global interactions (11). Despite these advances, pure Transformer-based approaches face a critical texture semantic gap in medical imaging (12). Unlike natural images, where objects are defined by distinct shapes, histological patterns are often defined by high-frequency textures and subtle boundary conditions. Transformers, which generally act as low-pass filters, tend to prioritize global shape over local texture, resulting in over-smoothed boundaries that fail to capture the precise (13), jagged edges of high-grade tumors.

Current hybrid methodologies attempt to bridge this gap by combining CNN encoders with Transformer bottlenecks (14). However, most existing solutions rely on naive feature fusion strategies, such as simple concatenation or summation, which treat texture (CNN features) and global context (Transformer features) as independent information streams (15). This approach fails to leverage the complementary nature of these modalities. We hypothesize that for optimal segmentation, texture should not merely be an additive feature but a guiding force. Specifically, the high-frequency morphological details captured by LBP should explicitly condition the attention mechanism, directing the Transformer to focus on texture rich regions such as glandular borders and nuclear clusters. Furthermore, the standard upsampling processes in decoder networks often degrade spatial precision, necessitating advanced feature aggregation modules that can reconstruct lost positional information and suppress stromal noise.

In this work, we propose a novel, texture guided hierarchical architecture designed to overcome the specific challenges of Gleason grading and glandular segmentation. Our approach fundamentally rethinks the interaction between handcrafted priors and deep learning mechanisms. Rather than discarding traditional descriptors, we integrate LBP directly into the query projection of the Swin, creating a attention mechanism that is sensitive to both global context and local morphological nuance. To ensure robust feature fusion, we introduce the DP-SFE module, which acts as a co-attention bridge to dynamically recalibrate features and mitigate the boundary blurring associated with patch merging. In the decoder, we depart from standard convolutions, employing ACB to reinforce directional structural edges and a CDFA to explicitly reconstruct positional coordinates.

The primary contributions of this paper are summarized as follows:

1. We propose a novel architecture where Swin Transformer queries are conditioned directly on LBP features. This ensures the attention mechanism is not just spatially aware but explicitly texture-sensitive, allowing the model to capture high-frequency morphological details often missed by standard global attention.
2. To resolve the misalignment between global context and local texture, we introduce the DP-SFE module. Acting as a co-attention bridge, it dynamically recalibrates feature responses to mitigate boundary blurring from patch merging, ensuring that local noise and global structure are not treated with equal importance.
3. We incorporate ACB to enhance feature extraction without the computational cost of large kernels. Unlike standard square kernels, the ACB employs directional convolutions to explicitly reinforce horizontal and vertical structural edges, which is critical for distinguishing dense object boundaries in segmentation.
4. We implement the CDFA to reconstruct precise positional information and suppress stromal noise during encoder-decoder fusion. These spatially coherent features are then aggregated by the Refined Segmentation Head (RSegH) to generate high-quality segmentation maps with sharp accurate boundaries.

## 2 Literature review

### 2.1 Clinical context and the need for automation

The Gleason system stratifies prostate cancer based on the architectural patterns of glands, currently reported as ISUP grade groups ranging from 1 (Gleason ≤3+3) to 5 (Gleason 9–10) (16). Clinical prognosis and treatment decisions depend not only on the presence of high-grade patterns but also on the spatial distribution and percentage of Gleason patterns 4 and 5 within a lesion (17). However, substantial inter and intra observer variability particularly at the critical 3/4 boundary has motivated the development of quantitative, segmentation based approaches on WSI to objectively delineate and grade tumor areas (18). Recent studies on deep learning architectures for prostate cancer analysis have explored these approaches in greater detail Table 1.

**Table 1:** Recent studies on deep learning architectures for prostate cancer analysis.

Method	Task & data	Key findings
Hierarchical ViT + Transformer Classifier (30)	Predict ERG/PTEN status from H&E WSIs	AUC 0.86–0.91 (ERG); illustrates ViT’s ability to model complex molecular morphology.
Domain-Agnostic CNN + Active Learning (18)	Epithelial segmentation & grading	κ=0.92 (internal), κ=0.96 (external); robust cross-site performance.
Transformer-MIL + GCN (31)	Weakly supervised grading (PANDA, TCGA)	SOTA performance using slide-level labels; efficient discriminative patch mining.
ViT on WSI Patches (32)	Patch classification & slide scoring	Validated viability of pure ViT pipelines for Gleason pattern aggregation.
Swin-Transformer + UNet Hybrid (33)	3D Multi-organ segmentation	Hybrids outperform pure CNNs/Transformers; validates hybrid approach for medical segmentation.
Dual-path Swin-Unet + MIM (34)	Gland segmentation (PANDA, SICAPv2)	mDice 0.947 (PANDA); establishes segmentation as key for interpretable grading.
Swin U-Net (34)	Prostate Gland Segmentation (PANDA, SICAPv2)	Dice 78.2 % precise malignant segmentation
Transformer Encoder + MLP Decoder (35)	Joint tumor segmentation & grading	Dice 97.5%; 96.6% grading accuracy; unified framework for dual tasks.
Hierarchical ViT with Attention Aggregation (36)	Grading on PANDA + clinical cohorts	κ=0.916; superior generalization across diverse external cohorts.
Ensemble Transfer-Learning CNNs (37)	Gleason classification on unlabeled WSIs	Outperformed patch-based models using only slide-level labels; high WSI-level accuracy.
Multiscale Fourier Transformer MIL (38)	Slide-level grading (RP + Biopsies)	AUC 0.88 overall; exceptional detection of Grade Group 5 (AUC 0.977).
Ensemble CNN + Transformer (39)	Patch Slide-level grading (Low-res 10×)	99.6% binary accuracy; proves cost-effectiveness of low-resolution analysis.

### 2.2 The era of handcrafted features

Early computer aided systems relied on handcrafted texture features, such as LBP and gray-level co-occurrence matrices, to represent glandular architecture. Radiomic studies utilizing these descriptors demonstrated that micro-texture is strongly associated with Gleason grade, distinguishing clinically significant (Gleason ≥ 7) from insignificant disease with accuracies of 80%–85% and AUCs of 0.80–0.85 (19). While classical classifiers using LBP-type features could achieve high classification accuracy, they often required accurate prior gland detection and struggled in regions of poorly formed pattern 4 where glandular structure is lost (20). These limitations specifically the manual feature design, sensitivity to noise and stain variation, and difficulty modeling complex fused glands necessitated a shift toward learned features.

### 2.3 Convolutional neural networks (CNNs)

CNNs overcame the rigidity of handcrafted pipelines by enabling end-to-end learning directly from image patches (21). Lucas et al. (22) demonstrated that retraining Inception-v3 (23) for pixel-wise classification aggregated into biopsy-level grade groups with ∼90%–92% accuracy (κ = 0.70), significantly outperforming earlier handcrafted methods in detecting pattern 4. Subsequent large-scale diagnostic systems combined segmentation and grading to achieve pathologist-level performance. Bulten et al. (24) developed a fully automated system that achieved a quadratic κ of 0.918 on a test set of 550 biopsies, outperforming most individual pathologists. Similarly, Ström et al. (25) trained deep networks on the population-based STHLM3 study, reaching an AUC of 0.997 for benign vs. malignant detection and a mean pairwise κ of 0.62 for grading.

Recent CNN research has focused on precise pattern-specific segmentation to address the subjectivity of manual quantification. Singhal et al. (18) utilized domain-agnostic training and deep active learning to generate pixel-level Gleason overlays with high internal accuracy (89.4%) and robust external performance. Chen et al. (17) developed segmentation models for pattern 3 and 4 glands to quantitatively estimate %GP4, a metric strongly associated with biochemical recurrence risk. Ensembles of EfficientNetB2, U-Net (7), and residual attention U-Net have further improved the semantic segmentation of stroma, benign tissue, and Gleason patterns, achieving clinically acceptable Dice and IoU scores (26). Integrated frameworks combining patch-level classification with grade-specific segmentation (e.g., Self-ONN-DeepLabV3) have reported κ scores above 0.92 for final grade group assignment (27). Furthermore, these segmentation concepts have been extended to alternative modalities, including stimulated Raman scattering microscopy (28) and prostate MRI (29).

### 2.4 Transition to transformers and attention mechanisms

Despite their success, CNNs possess intrinsic limitations: their local receptive fields make modeling long-range context difficult, and they remain sensitive to domain shifts in stain and scanner characteristics (40). This has driven a major trend toward attention mechanisms, transformers, and graph-based models capable of capturing global context in gigapixel WSIs. Lightweight CNNs augmented with hierarchical multi-attention modules have shown that attention-guided residual blocks focus learning on diagnostically salient structures (41). Hybrid CNN-Transformer designs now dominate the state-of-the-art. For instance, hybrid U-Nets coupling ResNet encoders with ViT blocks achieve superior performance on datasets like SICAPv2 while incorporating explainable AI tools for interpretability (42). Similarly, SegFormer-based models with MLP decoders or EdgeNeXt backbones successfully perform joint semantic segmentation and classification of Gleason patterns 3–5 (35). Beyond grid-based approaches, graph neural networks operating on patch-graphs utilize focal loss to improve the recognition of minority grade 5, outperforming several baselines (43). Zaheer et al. (14) extend U-Net into a transformer-attention hybrid (TAH-U-Net), merging ResNet50 convolutions with Vision Transformer blocks and attention guided skip connections; on SICAPv2 this yields gains of 4.6% Dice and 5.1% IoU over standard U-Net, while incorporating XAI tools such as LIME and occlusion sensitivity to support clinical trust. For volumetric imaging, Yu et al. (44) introduce a serial parallel CNN-Transformer with cross-window self-attention and multi-scale local enhancement, improving prostate MRI segmentation across multiple 3D datasets compared with six popular baselines. Mittmann et al. (45) focus on interpretable Gleason pattern segmentation by training on pathologist defined textual pattern descriptions with soft labels, achieving robust Gleason pattern maps that mirror expert reasoning. Complementing these segmentation centric works, Yang et al. (46) employ nnU-Net for automated prostate MRI segmentation as a backbone for radiomics and “habitat” analysis to predict high-grade Gleason scores and bone metastases, reaching AUCs around 0.90 in machine-learning classifiers.

## 3 Methodology

### 3.1 Overall architecture

The proposed architecture processes the input image through four progressive stages to extract robust features for prostate cancer segmentation (Figure 1). In Stage 1, the image passes through LBP1 and Swin-Transformer Block 1, followed by patch merging and fusion in DP-SFE Block 1. Stage 2 utilizes the output from DP-SFE Block 1, passing it through LBP2 and Swin-Transformer Block 2 where LBP2 features are concatenated with the query vector (Q) for texture guided attention before undergoing patch merging and refinement in DP-SFE Block 2. Stage 3 replicates this pattern with LBP3 and Swin-Transformer Block 3 feeding into DP-SFE Block 3, while in Stage 4, the output is processed by LBP4 and Swin-Transformer Block 4, with their outputs concatenated to form the final encoder feature map. To preserve spatial details, features from the first three encoder stages are passed through Asymmetric Convolution Blocks (ACB), which serve as refined skip connections. These ACB refined features are fused with upsampled maps from the preceding decoder stage via Cross-Directional Feature Aggregator (CDFA) modules , which utilize coordinate-aware attention to reconstruct spatial information. Finally, the Refined Segmentation Head (RSegH) takes the output from all CDFA modules, performs element-wise summation to aggregate multi-scale features, and adjusts the channel dimensions via a two-layer convolution block to generate the final semantic segmentation map.

**Figure 1:**
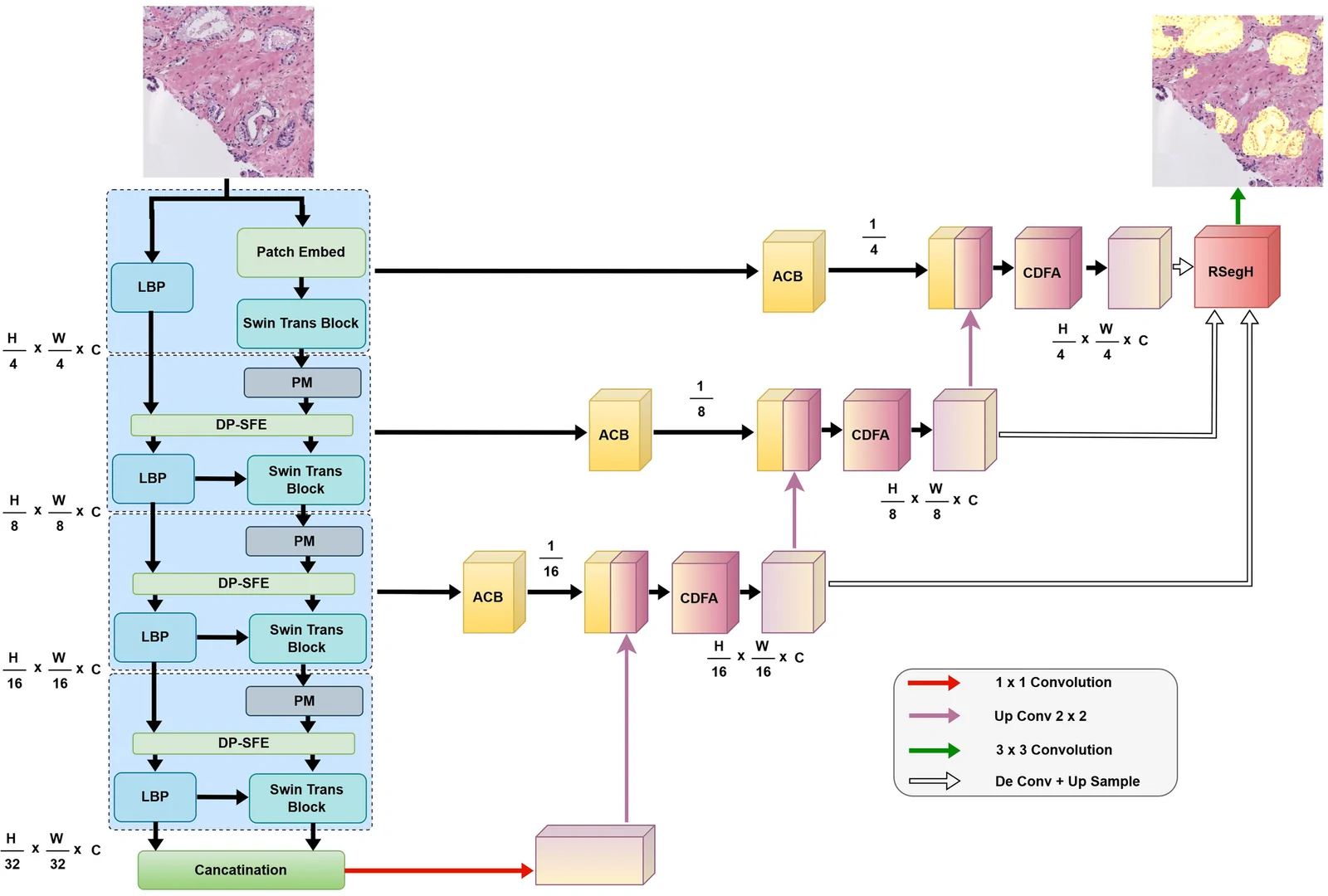
TexTNet architecture.

### 3.2 Attention computation

In the Vision Transformer (ViT) (9), Multi-Head Self-Attention (MSA) is used to capture global dependencies in the input feature map. However, the high computational complexity of this approach has led to the development of more efficient alternatives. One such alternative is the Swin Transformer (47), which reduces the computational complexity from $O(N^{2})$ to O(N), making it more suitable for general vision tasks, especially in large-scale datasets.

For a given input feature map $X\in R^{H\times W\times C}$, attention is computed by dividing the feature map into non-overlapping windows of size M×M. Each window operates independently, and the attention mechanism for each window is calculated sequentially. The first step in this process is projecting the input feature map X into queries (Q), keys (K), and values (V) through learnable weight matrices. This is expressed as Equation 1:

$$Q=XW_{Q},\;K=XW_{K},\;V=XW_{V}$$ (1)

where $W_{Q}$, $W_{K}$, and $W_{V}$ are the learnable weight matrices that project the input feature map X into their respective query, key, and value vectors.

Next, attention is computed by calculating the attention scores $A_{w}$ within each window. The attention score is computed as follows (Equation 2):

$$A=\text{Softmax}\left(\frac{QK^{⊤}}{\sqrt{d}}\right)V$$ (2)

where $A\in R^{M^{2}\times d}$ represents the attention score matrix, and $M^{2}$ is the number of patches (tokens) within the window. The Softmax function normalizes the unnormalized attention scores into a probability distribution, allowing the model to assign higher attention weights to more relevant tokens and lower weights to less relevant ones. This ensures that the model focuses on the most relevant regions of the feature map.

While the window-based self-attention mechanism works efficiently within local regions, it has a limitation in that it does not model long-range dependencies across the entire image. To overcome this limitation, a shifted window partitioning approach is introduced. This method alternates between two different partitioning configurations in consecutive Swin Transformer blocks. In the first configuration, the feature map is partitioned into non-overlapping windows, while in the next block, the windows are shifted by $\left(\lfloor \frac{M}{2}\rfloor ,\lfloor \frac{M}{2}\rfloor \right)$ pixels to create cross-window connections. This shifted window approach allows the model to capture long-range dependencies across windows while maintaining computational efficiency by only processing local windows.

In stage 2, after the attention computation and shifted window partitioning, a patch merging operation is applied. The patch merging step reduces the spatial resolution of the feature map, improving computational efficiency while enhancing the receptive field. The operation aggregates information from neighboring patches, allowing the model to capture more global context. The patch merging operation is performed by concatenating features from neighboring patches and applying a learnable linear transformation to reduce the spatial resolution and merge the patches. This can be expressed as Equation 3:

$$X_{\text{m}}=\text{Concat}\left(X_{1},X_{2},\ldots ,X_{n}\right)W_{\text{m}}$$ (3)

where $X_{i}\in R^{M\times M\times C}$ are the non-overlapping patches (tokens) in the feature map, and $W_{\text{m}}\in R^{C^{\prime }\times C}$ is the learnable weight matrix that projects the concatenated features into a new space. $C^{\prime }$ represents the output dimension after the merging process. The concatenation operation combines the features from neighboring patches, and the learned weight matrix $W_{\text{m}}$ reduces the number of channels, downsampling the feature map. The resulting merged feature map $X_{\text{m}}$ is then passed to the next processing block, such as the DP-SFE blocks (i.e., DP-SFE B1, DP-SFE B2, and DP-SFE B3), for further refinement.

While the Swin Transformer excels at modeling global dependencies, it lacks an explicit mechanism to capture high-frequency local variations critical for histopathology. To address this, new attention mechanism modifies the standard attention flow. By transforming and concatenating outputs from specific LBP blocks (e.g., LBP B-2, LBP B-3) directly with the query vector Q inside the Swin Transformer blocks, the attention scores become both spatially aware and texture-sensitive. This integration forces the model to focus on fine-grained morphological details of the tissue structure during segmentation.

### 3.3 Local binary pattern (LBP) texture descriptor

To generate these critical texture features for concatenation with the attention queries, we employ the LBP descriptor (5). In our four-stage architecture, LBP acts as a parallel feature extractor (blocks B-1 through B-4) that complements the Swin Transformer. By explicitly modeling local intensity changes, the LBP modules capture the chaotic nuclear arrangements and stromal textures characteristic of different Gleason grades. These texture maps are then fused with the Transformer’s query vectors or merged via DP-SFE modules, ensuring that the final segmentation map preserves high-frequency boundary details that deep learning backbones often smooth out.

For a given image $X\in R^{H\times W\times C}$, where H, W, and C represent the height, width, and the number of channels of the input image, we proceed to extract texture features using the LBP method. In LBP, a pixel is compared to its eight surrounding neighbors in a small window, typically a 3 × 3 block. The goal is to calculate a binary pattern for each pixel by comparing the pixel value to its neighbors. The function S (Equation 4) is used to determine whether each neighboring pixel has a greater or smaller intensity than the central pixel. This function is defined as:

$$S(p,c)=\left\{ \begin{array}{cc} 1, & \text{if}\;g(p)\geq g(c),\\ 0, & \text{if}\;g(p)<g(c), \end{array}\right.$$ (4)

where g(p) and g(c) represent the intensity values of the neighboring pixel p and the central pixel c, respectively. The function S produces a binary output for each comparison.

Each of the neighboring pixels (p_1, p_2, …, p_8) is compared to the central pixel, and a binary code is generated for each comparison. The 8-bit LBP (lbp(c)) for the central pixel (c) is computed as Equation 5:

$$\text{lbp}(c)=\sum_{i=1}^{8}2^{i-1}S(p_{i},c)$$ (5)

This results in an 8-bit binary number representing the local texture feature for the central pixel (c).

Once the LBP value is computed for each pixel in the image, the next step is to compute the histogram of LBP values for each local region (typically a 3×3 block). The histogram captures how often each unique LBP pattern occurs within the region. This histogram can be represented as Equation 6:

$$H_{\text{LBP}}=\left[\sum_{\;p\in P}S(p,c)\right]$$ (6)

where (P) represents the set of neighboring pixels, and (c) is the central pixel. The histogram (H_LBP_) summarizes the distribution of LBP values in the region.

After calculating the LBP histograms for all local regions in the image, the histograms are concatenated to form a global feature representation (Equation 7). This concatenation combines the local texture information into a single feature vector that describes the image’s overall texture pattern:

$$X_{\text{t}}=\text{Concat}(H_{\text{LBP}}^{1},H_{\text{LBP}}^{2},\ldots ,H_{\text{LBP}}^{N})$$ (7)

where N is the number of regions (or patches) in the image, and each $H_{\text{LBP}}^{i}$ is the histogram for a particular region. The resulting vector $X_{\text{t}}$ serves as the complete LBP feature map, providing a comprehensive representation of the local texture information in the image. This texture-driven representation is particularly advantageous for the attention mechanism, as standard self-attention often overfits to stain-specific color intensities. By conditioning the attention on $X_{\text{t}}$, we force the model to focus on invariant structural morphology such as nuclear density and epithelial roughness rather than variable chromatic values, ensuring robust generalization across diverse staining protocols.

However, while $X_{\text{t}}$ effectively encodes high-frequency textural details such as nuclear boundaries and stromal density, it lacks the long-range semantic context required to understand the overall glandular architecture. Conversely, the features extracted by the Swin Transformer, denoted as $X_{\text{m}}$, capture robust global dependencies but may suffer from boundary blurring due to the patch-merging process. Simply concatenating these disparate feature representations is suboptimal, as it treats local noise and global structure with equal importance. To resolve this semantic gap, we employ the DP-SFE module. This module acts as a co-attention bridge, dynamically recalibrating the feature response of the texture stream based on the semantic context of the transformer stream (and vice versa) before fusion occurs.

### 3.4 Dual path squeeze-fusion-excitation (DP-SFE)

The overview of the proposed DP-SFE co-attention module is shown in Figure 2. Unlike standard self-attention mechanisms that operate on a single data stream, DP-SFE treats the texture feature map $X_{\text{t}}$ (from the LBP module) and the semantic feature map $X_{\text{m}}$ (from the Swin Transformer) as a dual-stream input system.

**Figure 2:**
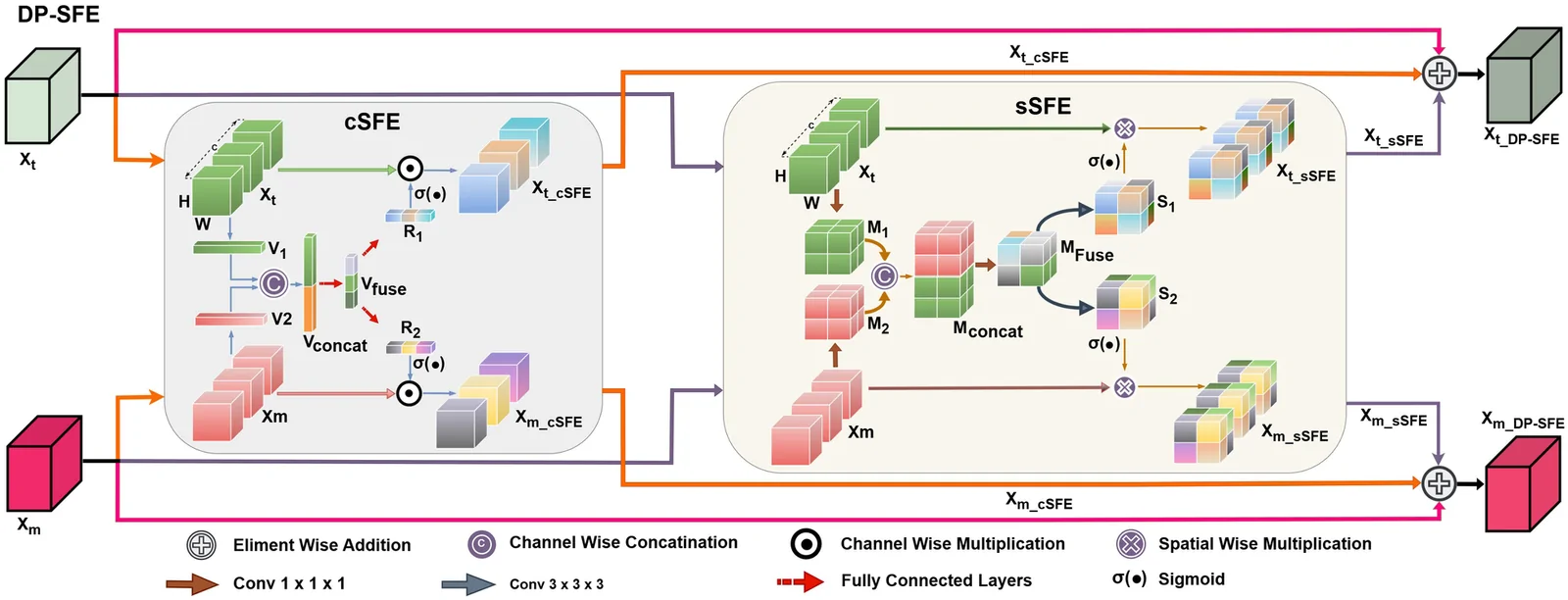
Dual path squeeze-fusion-excitation.

#### 3.4.1 Channel-wise squeeze-fusion-excitation (cSFE)

In the cSFE branch, the goal is to identify which feature channels are most discriminative across different staining protocols. The two feature maps $X_{\text{t}}$ and $X_{\text{m}}\in R^{H\times W\times C}$ are first compressed into global descriptors $V_{1}$ (Equation 8) and $V_{2}\in R^{C}$ (Equation 9) using global average pooling. This operation aggregates spatial information to generate a channel-wise statistic, computed as follows:

$$V_{1}(c)=\frac{1}{H\times W}\sum_{i=1}^{H}\sum_{\;j=1}^{W}X_{\text{t}}(i,j,c)$$ (8)

$$V_{2}(c)=\frac{1}{H\times W}\sum_{i=1}^{H}\sum_{\;j=1}^{W}X_{\text{m}}(i,j,c)$$ (9)

where H, W, and C refer to the height, width, and channel numbers of $X_{\text{t}}$ and $X_{\text{m}}$. Then, $V_{1}$ and $V_{2}$ are channel-wise concatenated and fed into a fully connected layer with weights $w\in R^{C\times 2C}$ and bias $b\in R^{C}$ to produce a fused vector $V_{\text{\textbackslash{},fuse}}\in R^{C}$ (Equation 10). This step models the inter-dependencies between the texture and semantic streams:

$$V_{\text{\textbackslash{},fuse}}=w[V_{1},V_{2}]+b$$ (10)

where [⋅] refers to the channel-wise concatenation operator. Next, $V_{\text{\textbackslash{},fuse}}$ is input to two separate fully connected layers to generate two channel-wise recalibration weights $R_{1}$ (Equation 11) and $R_{2}\in R^{C}$ (Equation 12). These weights determine the importance of each channel for the respective streams:

$$R_{1}=w_{1}V_{\text{\textbackslash{},fuse}}+b_{1}$$ (11)

$$R_{2}=w_{2}V_{\text{\textbackslash{},fuse}}+b_{2}$$ (12)

where $w_{1}$, $w_{2}\in R^{C\times C}$ and $b_{1}$, $b_{2}\in R^{C}$ are the weights and biases of the two separate fully connected layers. $R_{1}$ and $R_{2}$ are then applied back to the two input streams using channel-wise multiplication, generating the channel recalibrated features $X_{\text{t}}(\text{cSFE})$ (Equation 13) and $X_{\text{m}}(\text{cSFE})$ (Equation 14):

$$X_{\text{t}}(\text{cSFE})=\sigma (R_{1})⊙X_{\text{t}}$$ (13)

$$X_{\text{m}}(\text{cSFE})=\sigma (R_{2})⊙X_{\text{m}}$$ (14)

where σ(⋅) is the sigmoid function and ⊙ refers to the channel-wise multiplication.

#### 3.4.2 Spatial squeeze-fusion-excitation (sSFE)

While cSFE focuses on *what* features are important, sSFE focuses on *where* the informative regions are located (e.g., distinguishing glandular boundaries from background noise). The two feature maps $X_{\text{t}}$ and $X_{\text{m}}\in R^{H\times W\times C}$ are squeezed using 1×1 convolutional operations with kernels $K_{\text{in1}}$ and $K_{\text{in2}}\in R^{1\times 1\times C}$ and biases $B_{\text{in1}}$ and $B_{\text{in2}}\in R^{1}$, generating two spatial attention maps $M_{1}$ (Equation 15) and $M_{2}\in R^{H\times W\times 1}$ (Equation 16) as:

$$M_{1}=K_{\text{in1}}⋆X_{\text{t}}+B_{\text{in1}}$$ (15)

$$M_{2}=K_{\text{in2}}⋆X_{\text{m}}+B_{\text{in2}}$$ (16)

where ⋆ denotes the convolutional operator. Then, $M_{1}$ and $M_{2}$ are channel-wise concatenated and fed into a 1×1 convolutional layer with a kernel $K_{\text{\textbackslash{},fuse}}\in R^{1\times 1\times 2}$ and a bias $B_{\text{\textbackslash{},fuse}}\in R^{1}$ to produce a fused volume $M_{\text{\textbackslash{},fuse}}\in R^{H\times W\times 1}$ as the spatial feature fusion given in Equation 17:

$$M_{\text{\textbackslash{},fuse}}=K_{\text{\textbackslash{},fuse}}⋆[M_{1},M_{2}]+B_{\text{\textbackslash{},fuse}}$$ (17)

Next, $M_{\text{\textbackslash{},fuse}}$ is refined by two separate 3×3 convolutional layers with kernels $K_{\text{out1}}$ and $K_{\text{out2}}\in R^{3\times 3\times 1}$ and biases $B_{\text{out1}}$ and $B_{\text{out2}}\in R^{1}$ to generate the spatial recalibration weights $S_{1}$ (Equation 18) and $S_{2}$ (Equation 19):

$$S_{1}=K_{\text{out1}}⋆M_{\text{\textbackslash{},fuse}}+B_{\text{out1}}$$ (18)

$$S_{2}=K_{\text{out2}}⋆M_{\text{\textbackslash{},fuse}}+B_{\text{out2}}$$ (19)

$S_{1}$ and $S_{2}$ are then applied back to the two input feature maps using spatial multiplication, generating $X_{\text{t}}(\text{sSFE})$ (Equation 20) and $X_{\text{m}}(\text{sSFE})$ (Equation 21):

$$X_{\text{t}}(\text{sSFE})=\sigma (S_{1})⊗X_{\text{t}}$$ (20)

$$X_{\text{m}}(\text{sSFE})=\sigma (S_{2})⊗X_{\text{m}}$$ (21)

where ⊗ denotes the spatial multiplication operator.

Finally, the module integrates the learned recalibrations with the original inputs to preserve the initial feature identity while enhancing relevant details. The channel-wise recalibrated features and the spatially recalibrated features are fused with the input features $X_{\text{t}}$ and $X_{\text{m}}$ via element-wise addition. This results in the refined DP-SFE outputs $X{\text{t}}_{\text{i}}$ (Equation 22) and $X{\text{m}}_{\text{i}}$ (Equation 23):

$$X{\text{t}}_{\text{i}}=X_{\text{t}}+X_{\text{t}}(\text{cSFE})+X_{\text{t}}(\text{sSFE})$$ (22)

$$X{\text{m}}_{\text{i}}=X_{\text{m}}+X_{\text{m}}(\text{cSFE})+X_{\text{m}}(\text{sSFE})$$ (23)

where $X{\text{t}}_{\text{i}}$ and $X{\text{m}}_{\text{i}}$ represent the refined feature maps for the current stage (e.g., $X{\text{t}}_{\text{1}},X{\text{t}}_{\text{2}}\ldots$). This residual addition ensures that both global context and local texture contribute effectively to the final representation, strengthening the model’s ability to handle stain variability.

Following the DP-SFE refinement, the architecture progresses through subsequent stages to further integrate texture features with the attention mechanism. In Stage 2, the outputs from DP-SFE Block 1 ($X{\text{t}}_{\text{i}}$ and $X{\text{t}}_{\text{m}}$) are processed by LBP2 and Transformer Block 2. To explicitly guide the attention mechanism with texture information, the output of LBP2 is projected into a new channel dimension $C_{q}$, matching the query vector Q of the transformer as shown in Equation 24:

$$X^{\prime }{\text{t}}_{\text{i}}=\text{FC}(X{\text{t}}_{\text{i}})$$ (24)

Next, the transformed feature $X^{\prime }{\text{t}}_{\text{i}}$ is concatenated with the query vector Q. Self-attention is then computed based on this texture-enhanced query vector $Q_{\text{c}}$ (using Equation 2), ensuring the transformer focuses on regions with significant textural relevance as shown in Equation 25:

$$Q_{\text{c}}=\text{Concat}(Q,X^{\prime }{\text{t}}_{\text{i}})$$ (25)

The resulting merged feature map $X_{\text{m}}$ (using Equation 3) is then passed into DP-SFE Block 2 along with the LBP2 output for the next level of refinement given in Equation 26:

$$X_{\text{m}}=\text{Merge}(X^{\prime }{\text{t}}_{\text{i}},Q_{\text{c}})$$ (26)

This process repeats for Stage 3. In the final Stage 4, to produce the definitive segmentation map, the outputs from LBP4 and Transformer Block 4 are directly concatenated rather than passed through another DP-SFE block, merging the most refined texture and semantic features for the final prediction.

### 3.5 Asymmetric convolution block (ACB)

To enhance the feature extraction capability of the network without incurring the computational cost of larger kernels, we incorporate an ACB based on the work of (48). In standard CNNs, square kernels (e.g., 3×3) are universally used to capture spatial context. However, for dense prediction tasks like segmentation, distinguishing object boundaries requires sensitivity to directional features.

As illustrated in Figure 3, the ACB is deployed in encoder stages 1, 2, and 3, as well as in the decoder. The block consists of three parallel branches: a standard 3×3 convolution to capture local context, and two asymmetric convolutions with 1×3 (horizontal) and 3×1 (vertical) kernels (Equation 27). This multi-branch design approximates a cross-shaped receptive field. While the square kernel aggregates information from the central neighborhood, the asymmetric kernels explicitly reinforce the response to horizontal and vertical structural edges. By summing the outputs of these branches, the network learns a richer representation that is robust to geometric variations in the input WSI patches. While the Swin encoder captures macro-directional layouts at the patch level (acting as a low-pass filter), the ACB in the skip connections acts as a pixel-level high-pass filter. By directly routing micro-directional gradients like sharp glandular edges to the decoder, the ACB bypasses patch-merging degradation and preserves fine boundaries without double-encoding positional bias.

**Figure 3:**
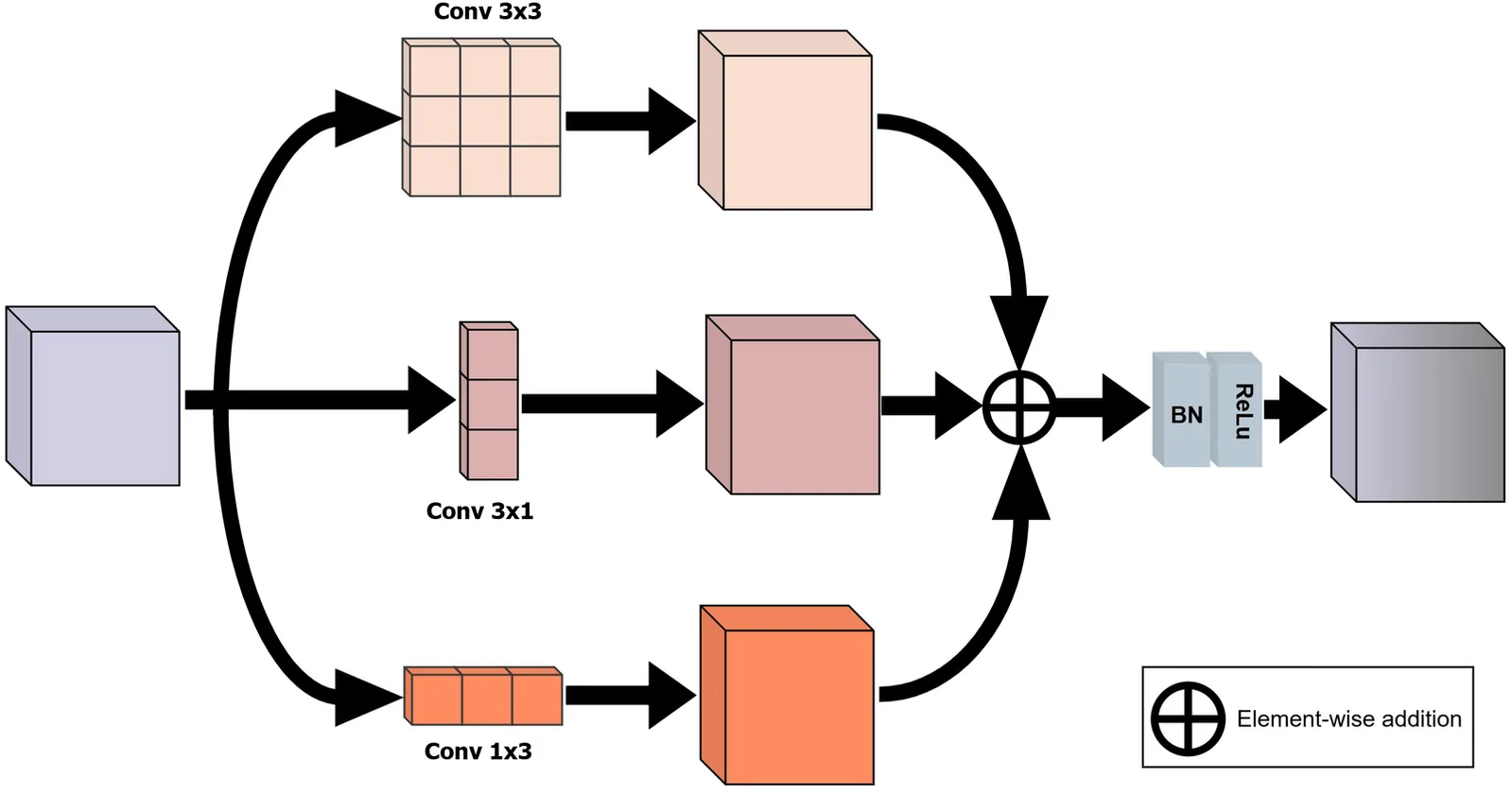
Asymmetric convolution block.

The fusion of these branches is performed via element-wise summation, followed by BN and a PReLU activation to introduce non-linearity while maintaining gradient flow. The mathematical formulation of the ACB is given as Equation 28:

$${\tilde{x}}_{i}=F_{3\times 3}(x_{i-1})+F_{3\times 1}(x_{i-1})+F_{1\times 3}(x_{i-1})$$ (27)

$$x_{i}=\sigma \left(\gamma_{i}\frac{{\tilde{x}}_{i}-E[{\tilde{x}}_{i}]}{\sqrt{\mathrm{Var}\;({\tilde{x}}_{i})+\epsilon_{i}}}+\beta_{i}\right)$$ (28)

where $x_{i}$ denotes the output feature map, $x_{i-1}$ is the input, and $F_{K_{h}\times K_{w}}$ represents a convolution operation with kernel size $K_{h}\times K_{w}$. The terms γ and β are learnable affine parameters of the BN layer, and σ(⋅) is the PReLU activation function.

In the decoder, the ACB plays a critical role in signal reconstruction. We attach an ACB immediately after each transposed convolution (upsampling) layer. Transposed convolutions often introduce high-frequency checkerboard artifacts due to uneven overlap of the kernel stride. The ACB mitigates this issue by smoothing the feature maps and enforcing spatial continuity, ensuring that the final segmentation masks possess clean, artifact-free boundaries.

### 3.6 Cross-directional feature aggregator (CDFA)

The transfer of features between the encoder and decoder provides essential detailed information for accurate segmentation and boundary recovery. However, simply fusing these features often introduces background noise from the stroma. To facilitate robust information fusion and reconstruct the precise positional information of prostate glands, we implement the CDFA module (Figure 4).

**Figure 4:**
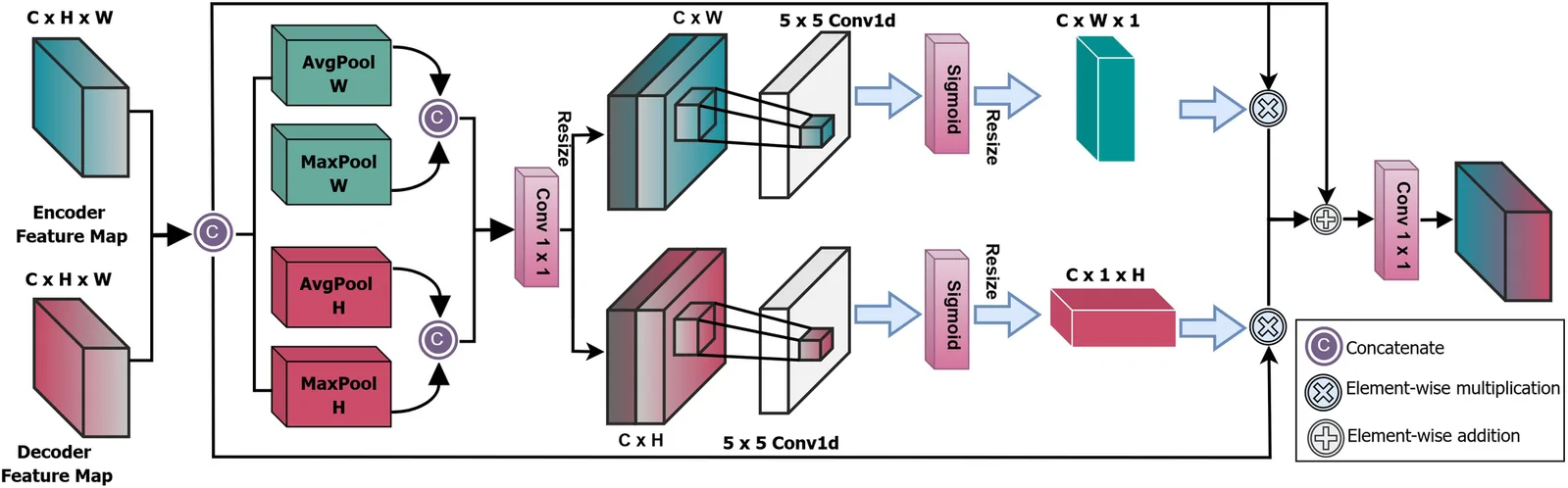
Architecture of cross-directional feature aggregator.

Unlike standard mechanisms, the CDFA is capable of determining the spatial coherence of glandular structures by exploiting the position invariance of image features along orthogonal directions. This allows the network to accurately locate regions of interest (ROI) and suppress the impact of stromal noise during the reconstruction phase.

Given the encoder output feature map $X_{1}\in R^{C\times H\times W}$ and the decoder output feature map $X_{2}\in R^{C\times H\times W}$, we first concatenate them along the channel dimension to form $M\in R^{2C\times H\times W}$. The tensor T is then reshaped to generate two distinct feature maps: $w\in R^{W\times C\times H}$ and $h\in R^{H\times C\times W}$, representing the vertical and horizontal spatial distributions, respectively. This operation is denoted as:

w,h←Resize(T) (29)

Subsequently, we apply average pooling and max pooling operations along the width dimension of w. This aggregates the spatial information into a height-focused vector. For each channel c and height index w, the outputs are:

$$x_{c}^{w}(w)=\frac{1}{W}\sum_{0\leq i<W}w_{c}(w,i)$$ (30)

$$y_{c}^{w}(w)=\max_{0\leq i<W}w_{c}(w,i)$$ (31)

where $x_{c}^{w}(w)$ and $y_{c}^{w}(w)$ represent the average-pooled and max-pooled features, respectively. Similarly, for h, we apply pooling along the height dimension to capture width-focused dependencies. For each channel c and width index h, the outputs are:

$${ℵ}_{c}^{h}(h)=\frac{1}{H}\sum_{0\leq j<H}h_{c}(j,h)$$ (32)

$$\eta_{c}^{h}(h)=\max_{0\leq j<H}h_{c}(j,h)$$ (33)

where ${ℵ}_{c}^{h}(h)$ and $\eta_{c}^{h}(h)$ denote the corresponding average-pooled and max-pooled features. The feature maps obtained from Equations 30–33 encapsulate sequential spatial information within their respective channels. We transform these into sequential representations for further processing. The features derived from w (Equation 34) and h (Equation 35) are separately concatenated and passed through a 2D convolution layer to adjust the channel dimensions:

$$z^{w}=\text{Conv2d}\left(\text{Cat}\left(x_{c}^{w},y_{c}^{w}\right)\right)$$ (34)

$$z^{h}=\text{Conv2d}\left(\text{Cat}\left(x_{c}^{h},y_{c}^{h}\right)\right)$$ (35)

where $z^{w}\in R^{1\times C\times W}$ and $z^{h}\in R^{1\times C\times H}$. These tensors are reduced in dimension to express sequential information effectively.

Compared to 2D convolutions, 1D convolutions are better suited for processing such sequential spatial data and are more computationally efficient. We employ 1D convolutions with a kernel size of 5 to enhance the fusion of positional information along the gland boundaries. Subsequently, 1D batch normalization is applied to achieve zero-mean, unit-variance normalization, followed by a Sigmoid activation to generate the attention weights for the width (Equation 36) and height (Equation 37) dimensions:

$$E^{w}=\sigma \left(\text{BN}\left(\text{Conv1d}(z_{w})\right)\right)$$ (36)

$$E^{h}=\sigma \left(\text{BN}\left(\text{Conv1d}(z_{h})\right)\right)$$ (37)

where σ denotes the Sigmoid activation function, and BN represents 1D batch normalization. The final output of the CDFA module is obtained by aggregating the original feature map T with its attention-weighted counterparts (Equation 38), ensuring that both horizontal and vertical spatial contexts contribute to the final segmentation:

$$\text{Output}=T⊕(T⊗E_{w})⊕(T⊗E_{h})$$ (38)

where ⊕ and ⊗ denote element-wise addition and multiplication, respectively.

### 3.7 RSegH module

Following the ACB and CDFA modules, the decoder generates multi-stage feature maps that encode a hierarchy of complementary spatial details and semantic representations. In histopathological Whole Slide Images (WSIs), reliable segmentation depends on the joint consideration of fine-grained cellular structures such as nuclei and glandular boundaries alongside macro-level tissue semantics like tumor and stromal regions.

Standard decoding strategies often overlook this multi-scale richness by relying solely on the final decoder output, a limitation that hampers the handling of the extreme resolution and complex morphology characteristic of prostate cancer. To bridge this gap, we introduce the Refined Segmentation Head (RSegH). This module systematically upsamples feature maps from all four decoder stages to a unified spatial resolution, enabling an element-wise summation that aggregates multi-level contextual information. The resulting fused representation is refined through a two-layer convolutional block to optimize channel dimensionality before generating the final pixel-level segmentation map.

## 4 Experimental details

### 4.1 Datasets

To comprehensively evaluate the robustness and generalization capability of our proposed framework, we utilize four diverse datasets: three Whole Slide Imaging (WSI) datasets and one Magnetic Resonance Imaging (MRI) dataset. We employ SICAPv2 (2), a comprehensive archive of H&E stained WSIs providing pixel-level annotations for Gleason patterns (G3, G4, and G5). Complementing this is the PANDA dataset (49) from the 2020 Grand Challenge, a large-scale multicenter WSI collection annotated with six classes ranging from benign tissue and stroma to Gleason grade 5. To validate functional tissue segmentation, we used the HuBMAP + HPA prostate subset (50) to demonstrate that our texture-guided attention mechanism successfully isolates fine cellular boundaries in healthy tissue, generalizing beyond malignant H&E patterns. Finally, to evaluate the model’s generalization on non-histological data, we include the ProstateX challenge dataset (51). This collection consists of retrospective prostate MRI exams from patients with elevated PSA levels and biopsy-confirmed diagnoses, serving as a benchmark for cross-domain diagnostic performance.

### 4.2 Implementation details

Rather than explicitly applying global stain normalization before feature extraction, our pipeline manages inter-site staining variance inherently. Specifically, the LBP descriptor (Equation 4) computes local textures based on relative pixel inequalities (g(p)≥g(c)), rendering the extracted features robust to absolute shifts in stain intensity. Furthermore, the DP-SFE module’s cSFE branch dynamically mitigates chromatic variations by recalibrating feature streams to emphasize stain-robust channels. All experiments were implemented using the PyTorch framework on NVIDIA RTX 3090 GPU. To ensure a fair and methodologically consistent comparison, all baseline models were retrained from scratch under the same experimental protocol, utilizing the identical 70/20/10 data split and seed 42. For WSI processing, patches were extracted at a resolution of 512×512 pixels. To mitigate edge artifacts during the spatial reconstruction phase, a 10% overlap was applied between adjacent patches. The experimental evaluation utilized a total of 18,783 patches for the SICAPv2 dataset and 9,408 patches for the PANDA Radboud subset. For the HuBMAP and ProstateX datasets, 351 and 1,962 patches were used, respectively. Model optimization was conducted using the Adam optimizer (52) with an empirically determined initial learning rate of $1\times 10^{-3}$ and a batch size of 8, utilizing a standard Swin-T (47) as the baseline integrated with UNet architecture for all comparative analyses. While the SICAPv2, HuBMAP, and PANDA datasets were trained for 100 epochs, the training duration for the ProstateX dataset was extended to 150 epochs to accommodate its specific morphological complexity. To enhance model robustness and prevent overfitting, data augmentation techniques including random flips, rotations, and shear transformations were applied during the training phase to expose the network to a wider range of histological variations. Finally, the segmentation performance was rigorously evaluated using the Dice Similarity Coefficient (DSC) and Intersection over Union (IoU) metrics to quantify the spatial accuracy of the predicted masks against the ground truth annotations.

### 4.3 Loss function

To optimize the model for multi-class Gleason grading in prostate tissue, we utilize a hybrid objective function that integrates the strengths of both pixel-wise classification and spatial overlap. The total loss, denoted as $L_{\mathrm{total}}$ (Equation 39), is formulated as the weighted sum of Multi-class Cross-Entropy ($L_{\mathrm{CE}}$) and Multi-class Dice Loss ($L_{\mathrm{Dice}}$), where $\lambda_{1}$ and $\lambda_{2}$ are empirical weighting coefficients:

$$L_{\mathrm{total}}=\lambda_{1}L_{\mathrm{CE}}+\lambda_{2}L_{\mathrm{Dice}}$$ (39)

The Cross-Entropy component provides stable gradients by penalizing the divergence between the predicted probability and the ground truth label across N pixels and C Gleason grades. For a multi-class problem, it is expressed as in Equation 40:

$$L_{\mathrm{CE}}=-\sum_{i=1}^{N}\sum_{c=1}^{C}y_{i,c}\log\;({\hat{y}}_{i,c})$$ (40)

where $y_{i,c}$ is a binary indicator of the ground truth class for pixel i, and ${\hat{y}}_{i,c}$ is the corresponding predicted probability for class c. This is complemented by the Multi-class Dice Loss, which is specifically designed to address the class imbalance inherent in histopathology datasets where certain Gleason patterns may occupy significantly smaller regions than others. The Dice loss is calculated as in Equation 41:

$$L_{\mathrm{Dice}}=1-\frac{2}{C}\sum_{c=1}^{C}\frac{\sum_{i=1}^{N}y_{i,c}{\hat{y}}_{i,c}+\epsilon }{\sum_{i=1}^{N}y_{i,c}+\sum_{i=1}^{N}{\hat{y}}_{i,c}+\epsilon }$$ (41)

In this formulation, ϵ serves as a smoothing factor (set to $1\times 10^{-5}$) to ensure numerical stability and prevent division by zero. By combining these two metrics, the model effectively learns to maintain high pixel-level accuracy while ensuring robust structural overlap for complex glandular morphologies, such as the poorly formed or fused glands characteristic of high-grade Gleason patterns.

## 5 Results

### 5.1 Comparison with SOTA methods

#### 5.1.1 Results on SICAPv2

We compare TexTNet with several state-of-the-art methods for prostate Gleason grade segmentation on the SICAPv2 dataset (Table 2). Each method was evaluated on accuracy (Acc), mean DSC (mDSC), and DSC scores for G3, G4, and G5. TransUNet integrates Transformers into the U-Net backbone, enhancing global context extraction. However, despite a robust design, its DSC for G3, G4, and G5 (86.95, 87.17, 86.33, respectively) lags behind TexTNet, particularly in G5, where fine-grained segmentation is crucial. The addition of a Transformer decoder improves segmentation performance, but it still struggles with local feature refinement, which is a key advantage of TexTNet. MFTrans, a multi-resolution fusion transformer, outperforms TransUNet in capturing both high- and low-magnification features, achieving DSC = 86.42 (G3), 86.85 (G4), and 86.10 (G5). While MFTrans is effective in handling multi-scale features, it does not match TexTNet’s performance in boundary precision and fine-grained segmentation, where TexTNet’s ACB and CDFA modules excel at capturing local textures and spatial coherence. DHUnet employs a dual-branch hierarchical architecture combining Swin Transformer and ConvNeXt modules. It achieves competitive performance, with DSC = 87.61 (G3), 87.90 (G4), and 87.15 (G5). However, the lack of specialized modules like TexTNet’s ACB for boundary refinement and CDFA for multi-scale feature fusion limits its ability to fully leverage the benefits of fine-grained segmentation in high-grade regions. CTNet combines CNNs and Vision Transformers with channel attention and cross-attention mechanisms, achieving a DSC = 88.13 (G3), 88.27 (G4), and 87.60 (G5).

**Table 2:** Performance comparison with SOTA on SICAPv2.

Method	mAcc	mDSC	DSC (G3)	DSC (G4)	DSC (G5)
TransUNet (53)	91.61 ± 0.64	86.82 ± 0.49	86.95 ± 0.71	87.17 ± 0.53	86.33 ± 0.68
MFTrans (54)	92.09 ± 0.41	86.46 ± 0.66	86.42 ± 0.52	86.85 ± 0.70	86.10 ± 0.47
DHUnet (55)	92.44 ± 0.69	87.56 ± 0.43	87.61 ± 0.61	87.90 ± 0.45	87.15 ± 0.58
CTNet (56)	93.62 ± 0.55	88.01 ± 0.63	88.13 ± 0.44	88.27 ± 0.67	87.60 ± 0.51
MaskHIT (57)	**94.36** ± 0.48	89.98 ± 0.54	90.03 ± 0.39	90.34 ± 0.59	89.56 ± 0.42
TexTNet	94.30 ± 0.28	**90.59** ± 0.31	**90.67** ± 0.25	**90.88** ± 0.29	**90.22** ± 0.34

Bold means highest value, underline means second highest value.

CTNet performs well, especially in handling multimodal data, but its focus on global feature extraction and attention may not be as effective as TexTNet in maintaining fine-grained local details, crucial for accurate Gleason grading. MaskHIT, using masked transformers for semi-supervised learning, achieved DSC = 90.03 (G3), 90.34 (G4), and 89.56 (G5), outperforming most models in terms of boundary delineation. The self-supervised learning used in MaskHIT helps mitigate data sparsity, but TexTNet still outperforms MaskHIT in G5, where multi-scale feature aggregation and local refinement are crucial. TexTNet’s ability to refine local features through ACB and CDFA gives it a distinct edge. Finally, TexTNet achieves the highest mDSC of 90.59 and superior DSC scores in all three Gleason grades, with 90.67 (G3), 90.88 (G4), and 90.22 (G5). This improvement can be attributed to the integrated ACB for local feature extraction, CDFA for multi-scale feature fusion, and RSegH for final feature aggregation. The qualitative analysis is given in Figure 5,

**Figure 5:**
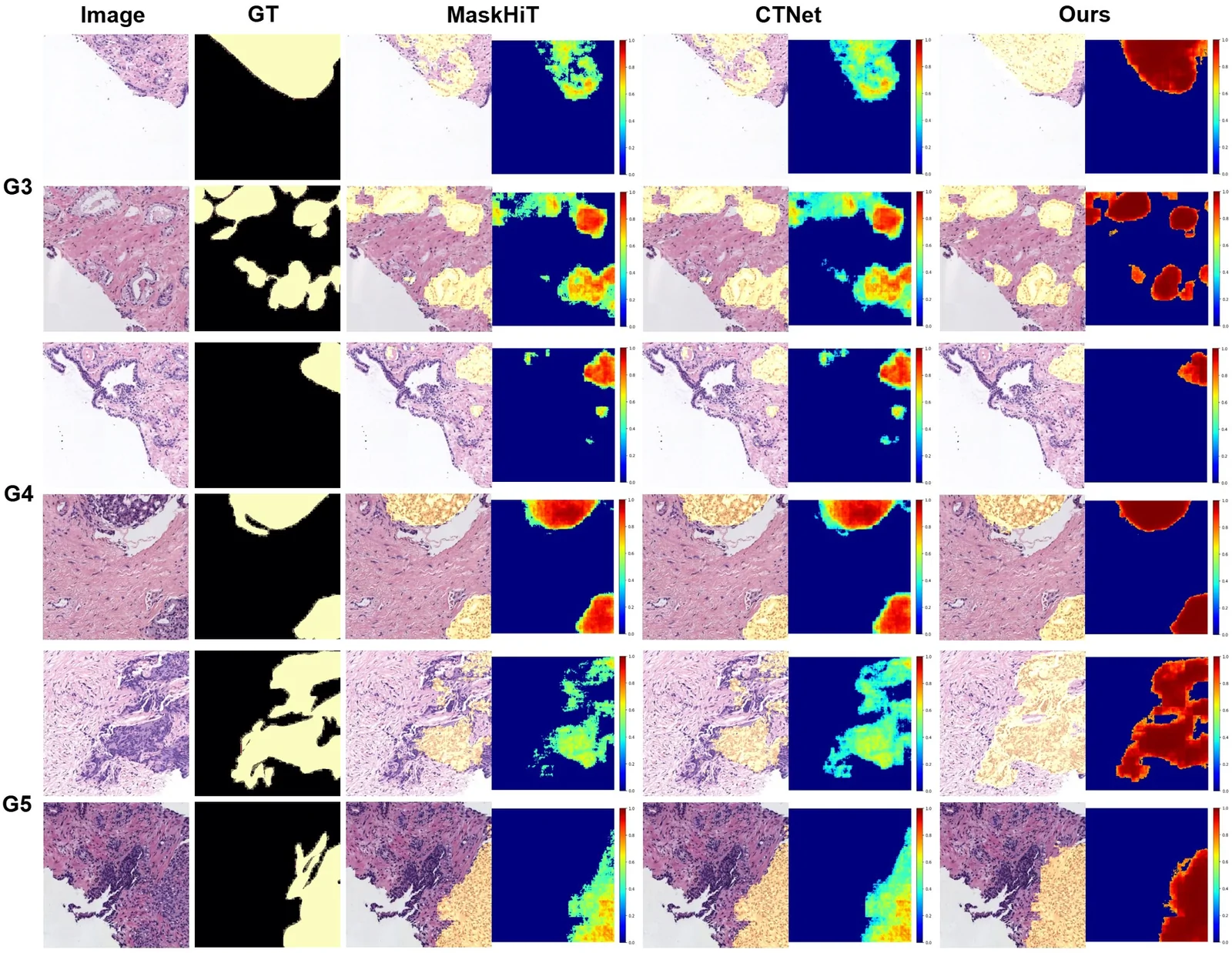
Qualitative comparison of TexTNet with SOTA methods on the SICAPv2 dataset.

#### 5.1.2 Results on PANDA

The performance of TexTNet also compared with several SOTA methods for prostate tissue segmentation on the PANDA dataset (Table 3). TransUNet achieved solid results but struggled with fine-grained details, especially in G5. MFTrans improved on this by incorporating multi-resolution features but still did not match TexTNet’s ability to refine local features and handle high-grade regions. DHUnet fused global and local features with its dual-branch architecture, but its performance was limited by its lack of dedicated local refinement, resulting in lower accuracy for G5 segmentation. CTNet excelled in multimodal tasks but fell short in precise G5 segmentation due to its lack of fine-grained local feature enhancement. MaskHIT utilized self-supervised learning, improving segmentation, especially with limited data. However, it lacked the multi-scale fusion and local feature refinement seen in TexTNet. TexTNet, with its combination of LBP-derived texture features and the global features from SwinT, achieved the best results with mDSC = 88.07 and DSC = 89.41 (G3), 88.14 (G4), and 86.66 (G5). The attention mechanism in TexTNet is texture-aware, as queries are conditioned on LBP features, ensuring fine-grained details are preserved, especially in regions with complex tissue structures like G5. The integration of ACB, CDFA, and RSegH further enhances performance, allowing for precise segmentation across all grades (Figure 6).

**Table 3:** Performance comparison with SOTA on PANDA.

Method	mAcc	mDSC	DSC (G3)	DSC (G4)	DSC (G5)
TransUNet (53)	90.37 ± 0.62	83.42 ± 0.55	84.65 ± 0.67	83.48 ± 0.48	82.12 ± 0.59
MFTrans (54)	91.46 ± 0.44	84.14 ± 0.61	85.23 ± 0.52	84.22 ± 0.65	82.98 ± 0.41
DHUnet (55)	91.28 ± 0.58	85.01 ± 0.39	86.31 ± 0.63	85.08 ± 0.51	83.64 ± 0.66
CTNet (56)	92.11 ± 0.53	85.97 ± 0.64	87.13 ± 0.46	86.02 ± 0.57	84.75 ± 0.38
MaskHIT (57)	92.81 ± 0.49	86.50 ± 0.42	87.79 ± 0.55	86.48 ± 0.36	85.23 ± 0.51
TexTNet	**92.95** ± 0.32	**88.07** ± 0.29	**89.41** ± 0.25	**88.14** ± 0.28	**86.66** ± 0.34

Bold means highest value, underline means second highest value.

**Figure 6:**
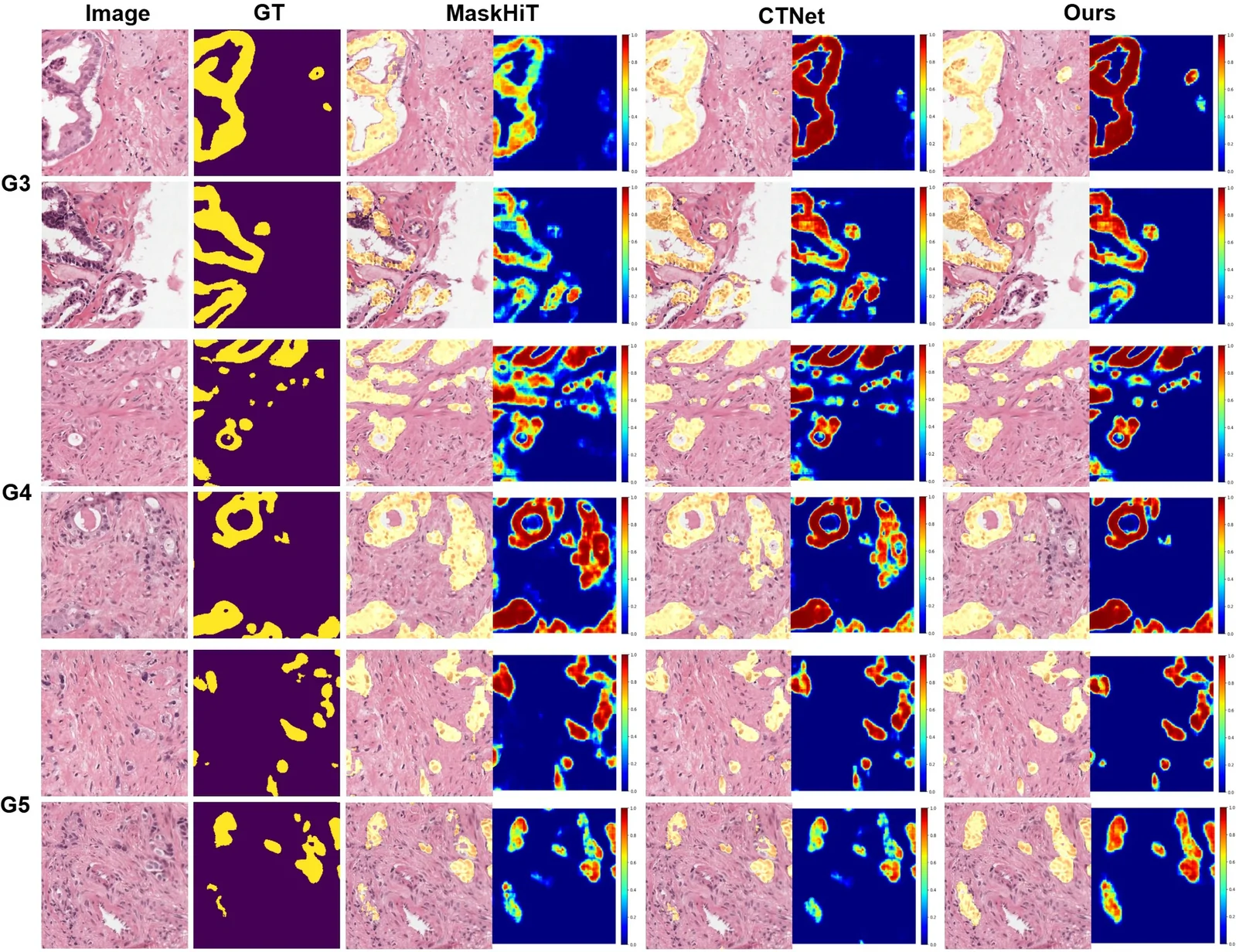
Qualitative comparison of TexTNet with SOTA methods on the PANDA dataset.

#### 5.1.3 Results on HuBMAP

To evaluate its performance in segmenting functional tissue units (FTUs) across various organs, TexTNet was compared with several SOTA methods on the HuBMAP dataset (Table 4), where the primary objective is to accurately identify and locate each FTU within multi-organ biopsy slides. TransUNet struggled to capture fine tissue details despite modeling global dependencies. MFTrans improved by incorporating multi-resolution fusion but lacks TexTNet’s texture-sensitive attention from LBP features. DHUnet, though benefiting from a dual-branch architecture, still falls short in refining local tissue features compared to TexTNet. CTNet performed well but struggled with detailed tissue structures, particularly at boundaries. MaskHIT excelled with self-supervised pretraining but lacks the multi-scale feature fusion and texture-aware attention of TexTNet. TexTNet (Acc = 97.40, DSC = 86.93) outperformed all methods, combining global feature extraction through Swin Transformer and local feature refinement via LBP attention, resulting in superior segmentation accuracy, particularly for complex tissue regions like FTUs. Furthermore the qualitative analysis is provided in Figure 7.

**Table 4:** Performance comparison with SOTA on HuBMAP.

Method	Acc	DSC
TransUNet (53)	94.69 ± 0.64	83.52 ± 0.58
MFTrans (54)	95.48 ± 0.47	84.37 ± 0.61
DHUnet (55)	95.82 ± 0.63	84.91 ± 0.52
CTNet (56)	96.56 ± 0.55	85.33 ± 0.67
MaskHIT (57)	96.12 ± 0.42	85.79 ± 0.59
TexTNet	**97.40** ± 0.38	**86.93** ± 0.29

Bold means highest value, underline means second highest value.

**Figure 7:**
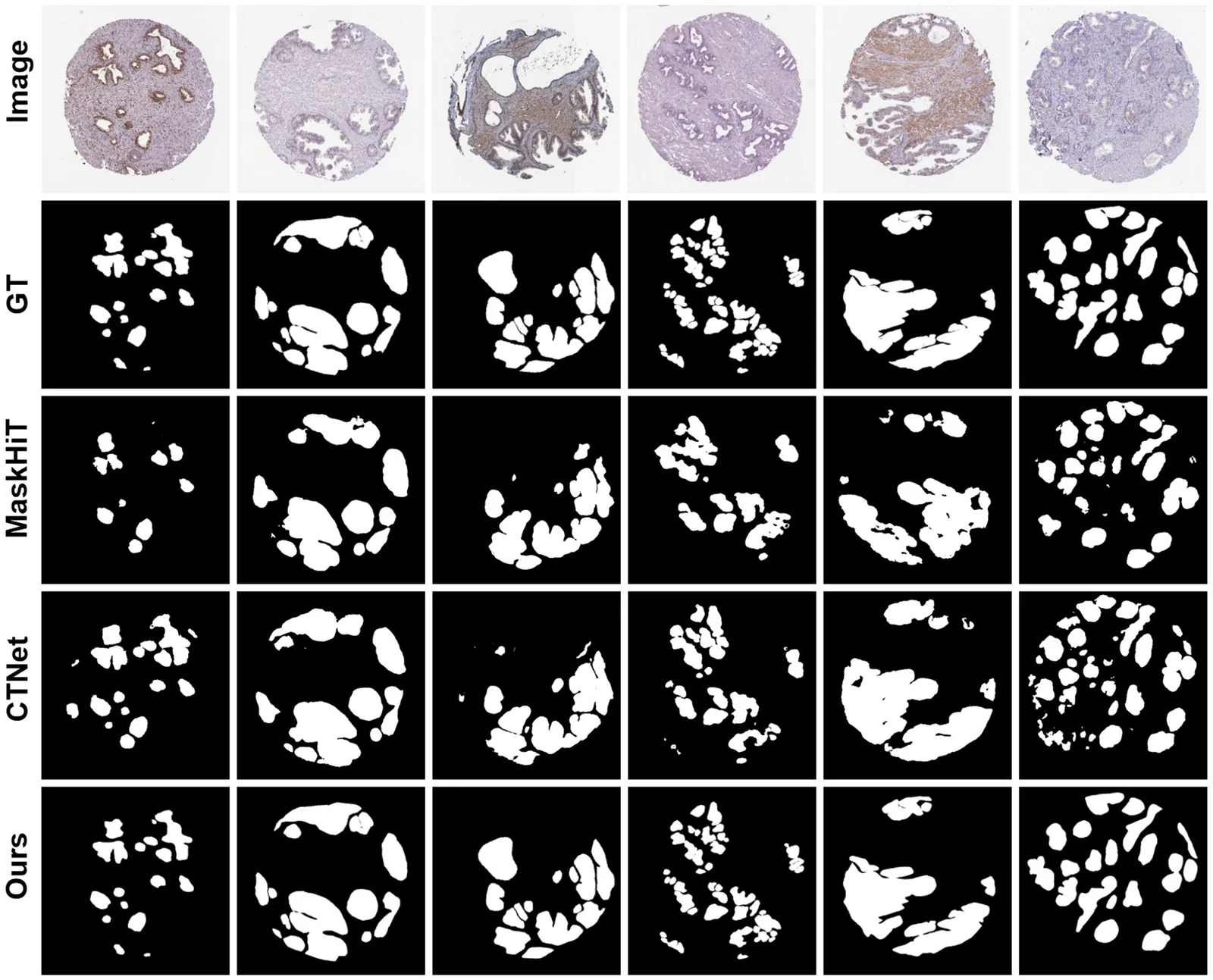
Qualitative comparison of TexTNet with SOTA methods on the HuBMAP dataset.

#### 5.1.4 Results on ProstateX

To show the generalizability of the model, TexTNet was further evaluated on the diverse ProstateX MRI dataset, where it demonstrated superior performance over existing methods by achieving higher segmentation accuracy and DSC scores across all regions of interest, particularly in the TZ, PZ, and Urethra regions (Table 5). Compared to TransUNet, MFTrans, and DHUnet, TexTNet showed notable increases in DSC: ↑ 1.46% for TZ, ↑ 1.18% for PZ, and ↑0.43% for Urethra. The LBP-guided attention in TexTNet plays a critical role in enhancing the model’s sensitivity to texture, which is particularly beneficial for segmenting fine-grained tissue structures, such as Urethra and PZ. While CTNet and MaskHIT also showed strong performance, TexTNet outperformed both methods due to its ability to integrate global context via Swin Transformer and refine local features with LBP-guided attention. This multi-scale feature fusion allowed TexTNet to better capture challenging tissue structures, especially in Urethra, resulting in ↑ 0.43% higher DSC compared to MaskHIT. The Swin Transformer’s global feature extraction, combined with ACB, CDFA, and RSegH, provided TexTNet with robust handling of both large-scale structures and fine details, contributing to significant improvements in the segmentation of complex regions. This made TexTNet the most accurate method for prostate zone segmentation across the ProstateX dataset. The qualitative analysis provided in Figure 8.

**Table 5:** Performance comparison with SOTA on ProstateX.

Method	mAcc	DSC(TZ)	DSC(PZ)	DSC(CZ)	DSC(AFS)	DSC(Urethra)
TransUNet (53)	96.86 ± 0.61	93.79 ± 0.54	88.30 ± 0.67	54.83 ± 0.49	51.44 ± 0.63	44.28 ± 0.57
MFTrans (54)	97.28 ± 0.44	94.12 ± 0.65	89.25 ± 0.52	57.62 ± 0.60	52.91 ± 0.41	46.68 ± 0.62
DHUnet (55)	97.83 ± 0.58	94.43 ± 0.43	89.72 ± 0.61	58.99 ± 0.54	54.13 ± 0.66	47.95 ± 0.48
CTNet (56)	98.17 ± 0.51	94.84 ± 0.59	90.09 ± 0.46	60.25 ± 0.67	55.67 ± 0.53	48.87 ± 0.42
MaskHIT (57)	**98.49** ± 0.42	95.11 ± 0.48	91.03 ± 0.39	61.34 ± 0.55	56.19 ± 0.47	49.06 ± 0.51
TexTNet	98.15 ± 0.29	**97.21** ± 0.35	**92.43** ± 0.26	**62.47** ± 0.21	**56.52** ± 0.26	**49.63** ± 0.29

Bold means highest value, underline means second highest value.

**Figure 8:**
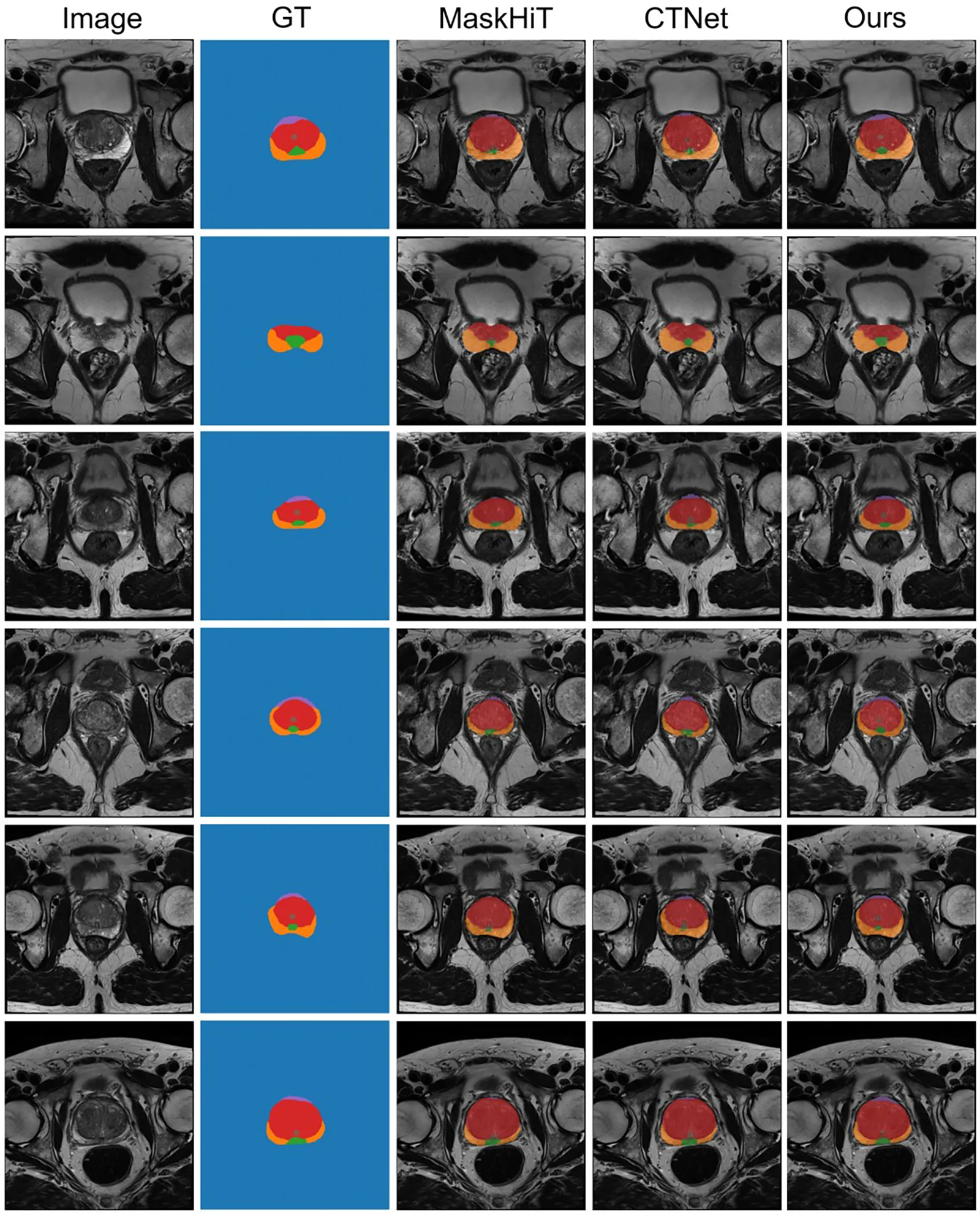
Qualitative comparison of TexTNet with SOTA methods on the ProstateX dataset.

The Table 6 presents a quantitative comparison of TexTNet with SOTA methods on several datasets, including SICAPv2, PANDA, HuBMAP, and ProstateX. TexTNet demonstrates competitive performance, achieving superior accuracy and DSC in most cases. Notably, TexTNet outperforms existing methods such as SS-ViT, DL-DN, PathTree-Ny, and others on key metrics, highlighting its effectiveness in prostate cancer detection and grading.

**Table 6:** Quantitative comparison with SOTA methods on key datasets.

Dataset	Method	Acc	DSC
SICAPv2	SS-ViT (58)	94.00	87.60
	DL-DN (59)	89.00	89.00
	SegFormer (60)	72.30	86.20
	Ours	94.30	90.59
PANDA	PathTree-Ny (61)	62.35	61.54
	EffNet-DLV3 (62)	81.14	84.90
	Att-LSTM (63)	69.12	67.45
	Ours	92.95	88.07
HuBMAP	UNeXt50(64)	–	83.60
	CoaT (65)	–	79.32
	SS-FTU (66)	–	83.87
	GlomNet (67)	–	86.74
	Ours	97.40	86.93
ProstateX	SPCT (68)	–	92.23
	HighRes3DNet (69)	–	89.00
	BBRT-Net (70)	–	89.27
	Ours	98.15	94.82

### 5.2 Ablation studies

To assess the contribution of each individual module in TexTNet, we conducted an ablation study on the SICAPv2 dataset (Table 7), focusing on the segmentation of prostate cancer Gleason grades (G3, G4, and G5). The study evaluates the structural impact of the ACB, CDFA, and RSegH modules on overall accuracy, mDSC, and specific grade-level segmentation performance. Starting with the baseline model, the integration of the ACB module in Model A yields a clear improvement in segmentation quality, raising the overall Accuracy to 92.25% and the mDSC to 87.11%. At the grade level, the DSC score increased by ↑ 1.49% for G3, ↑ 0.66% for G4, and ↑ 0.82% for G5. This confirms that ACB effectively enhances boundary refinement, particularly in low-grade regions, by optimizing local feature extraction and reinforcing structural edges where boundary precision is critical. In comparison, the CDFA module evaluated individually in Model B demonstrates an even stronger standalone performance, achieving a higher overall Accuracy of 92.68% and a higher mDSC of 88.38%. Specifically, CDFA boosts the baseline performance by ↑ 0.73% for G3, ↑ 0.49% for G4, and ↑ 0.45% for G5. This indicates that while ACB is highly capable of local contextual refinement, CDFA’s dense aggregation scheme is natively more proficient at capturing dense global-to-local feature streams and suppressing background noise across subtle intensity transitions. Crucially, when combining both modules in Model C, a powerful architectural synergy is observed rather than a conflict. Model C achieves a substantial leap to 94.04% Accuracy and 90.26% mDSC, showing a further grade-specific improvement of ↑ 0.53% for G3, ↑ 1.93% for G4, and ↑ 1.56% for G5. This underscores that ACB and CDFA complement each other effectively, successfully balancing fine local boundary details with robust multi-scale global context. Finally, the Proposed model which integrates ACB, CDFA, and the RSegH achieves the highest overall performance (94.30% Accuracy, 90.59% mDSC), providing an additional grade-level increase of ↑ 0.95% for G3, ↑ 0.57% for G4, and ↑ 0.51% for G5. The RSegH module successfully refines the final segmentation mask by aggregating multi-scale features and ensuring sharp boundaries, particularly in morphologically complex high-grade regions like G5.In conclusion, each module contributes distinct yet complementary advantages to the network: the ACB module excels at local boundary alignment, CDFA provides superior multi-scale feature fusion and spatial coherence, and RSegH ensures optimized final mask generation and fine-grained refinement.

**Table 7:** Ablation study evaluating the individual and collective impact of the proposed decoder modules (ACB, CDFA, and RSegH) on the SICAPv2 dataset using a fixed encoder backbone.

Configuration	ACB	CDFA	RSegH	mAcc	mDSC	Parameters(M)	GFLOPS	Inf. Time(s)
Baseline	×	×	×	90.63	86.18	27.42	11.81	0.0112
Model A	✓	×	×	92.25	87.11	27.85	12.35	0.0158
Model B	×	✓	×	92.68	88.38	28.14	15.17	0.0215
Model C	✓	✓	×	94.04	90.26	28.57	15.65	0.0231
Proposed	✓	✓	✓	94.30	90.59	29.83	16.80	0.0254

To evaluate the contribution of our principal encoder, we conducted an ablation study on the SICAPv2 dataset (Table 8). The baseline Swin Transformer yields an Accuracy of 90.63% and an mDSC of 86.18%. Integrating LBP features via naive fusion offers marginal gains (↑ 0.63% Accuracy, ↑ 0.65% mDSC), indicating that simple addition underutilizes texture data. Conversely, implementing our LBP-Guided Attention mechanism explicitly conditions the transformer on texture, resulting in a substantial performance leap to 93.18% Accuracy and 89.21% mDSC. Finally, incorporating the DP-SFE module effectively bridges local texture and global semantics, mitigating boundary blurring during patch merging. This full encoder configuration achieves the peak performance of 94.30% Accuracy and 90.59% mDSC.

**Table 8:** Ablation study of the encoder modules on SICAPv2.

Configuration	LBP (Naive)	LBP-Guided Attn	DP-SFE	mAcc	mDSC
Baseline (Swin Only)	×	×	×	90.63	86.18
Swin + LBP	✓	×	×	91.26	86.83
Swin + LBP-Guided Attn	×	✓	×	93.18	89.21
Proposed (Full Encoder)	×	✓	✓	94.30	90.59

We also conducted an ablation to evaluate the impact of different skip connection modules on prostate Gleason grade segmentation, specifically using the SICAPv2 dataset (Table 9). The U-Net skip connection (Identity), used in the baseline model, preserved spatial information but lacked feature enhancement. As a result, the model struggled with boundary precision, especially in G4 and G5, leading to suboptimal segmentation. The model achieved solid performance but was limited in fine-grained segmentation, particularly for high-grade regions. To establish a more rigorous baseline, we use a standard 3×3 convolution skip connection, which provided learnable feature refinement and improved local context aggregation (90.12% mDSC) compared to the raw identity mapping. However, this uniform square kernel remained limited in capturing the specific directional morphology of glandular boundaries. The Squeeze-and-Excitation (SE) module was then tested. It refined feature recalibration, improving the model’s attention on important regions, which boosted segmentation for subtle transitions in G3 and G4. However, SE didn’t improve boundary precision as effectively, leading to moderate improvement in accuracy (↑ 0.50%) and mDSC (↑ 0.63%). Then we incorporated the ACB (Proposed) module, which uses directional convolutions to refine boundaries by explicitly capturing horizontal and vertical edges. This enhancement was particularly beneficial for boundary refinement in high-density regions like G4 and G5. The model showed significant improvement (↑ 0.23% accuracy, ↑ 0.23% mDSC), demonstrating that ACB excels in local feature extraction and improves segmentation precision in challenging areas.

**Table 9:** Effectiveness of different skip connection modules on SICAPv2.

Skip connection	mAcc	mDSC
Standard U-Net skip (identity)	94.35	89.73
Standard 3×3 convolution	94.55	90.12
Squeeze-and-excitation	94.85	90.36
ACB (Proposed)	94.30	90.59

At last evaluated different upsampling techniques on the SICAPv2 dataset (Table 10) to assess their impact on prostate Gleason grade segmentation. Bilinear Interpolation achieved 88.46% accuracy and 85.67% mDSC. While efficient, this method struggles with preserving finer details, leading to less accurate segmentation in complex regions like G4 and G5. The Deconvolution method showed significant improvement, with ↑ 3.70% accuracy and ↑ 2.88% mDSC. It provides better feature resolution during upsampling, offering improved segmentation performance, particularly in areas with subtle texture variations. The CDFA (Proposed) method achieved the highest performance, with ↑ 2.14% accuracy and ↑ 2.04% mDSC. CDFA excels by aggregating multi-scale features, ensuring more precise segmentation, especially in regions like G5, where high detail is crucial. In conclusion, while Bilinear Interpolation is simple and efficient, it lacks the ability to preserve fine details. Deconvolution offers improved performance, but CDFA outperforms both methods, providing the most accurate segmentation by leveraging multi-scale feature fusion.

**Table 10:** Effectiveness of different upsampling modules.

Upsampling Method	mAcc	mDSC
Bilinear Interpolation	88.46	85.67
Deconvolution	92.16	88.55
CDFA (Proposed)	94.30	90.59

To evaluate the internal fusion strategy of the RSegH module, we conducted an ablation study comparing various multi-scale aggregation methods on the SICAPv2 dataset, as detailed in Table 11. The results indicate that element-wise summation is the most effective approach, likely because the feature maps are already semantically and spatially aligned by the preceding ACB and CDFA modules. This strategy achieves superior segmentation accuracy while ensuring computational efficiency compared to more complex learned-weighting or attention-gated alternatives.

**Table 11:** Comparison of internal fusion strategies for the RSegH module on SICAPv2.

Fusion strategy	mAcc	mDSC
Concatenation + 1 × 1 convolution	94.12	90.15
Learned scalar weighting (BiFPN-style)	94.21	90.42
Attention-gated multi-scale fusion	94.25	90.51
Element-wise summation (current)	94.30	90.59

To evaluate the training stability and convergence behavior of the proposed architecture, we analyzed the epoch-wise error deltas (the discrepancy between training and validation loss) against a zero-gap baseline. As illustrated in Figure 9, the distribution of the loss delta magnitudes clusters tightly around the zero mark, demonstrating that the network consistently maintains minimal divergence between training and validation performance across epochs. While consecutive epochs are not independent experimental observations, this tight clustering reflected by a Wilcoxon signed-rank test distribution (p<0.05) serves as a quantitative indicator of exceptional training stability. It confirms that the model optimizes smoothly without erratic fluctuations or severe overfitting spikes, thereby validating the architectural reliability of our texture-guided framework during the training phase.

**Figure 9:**
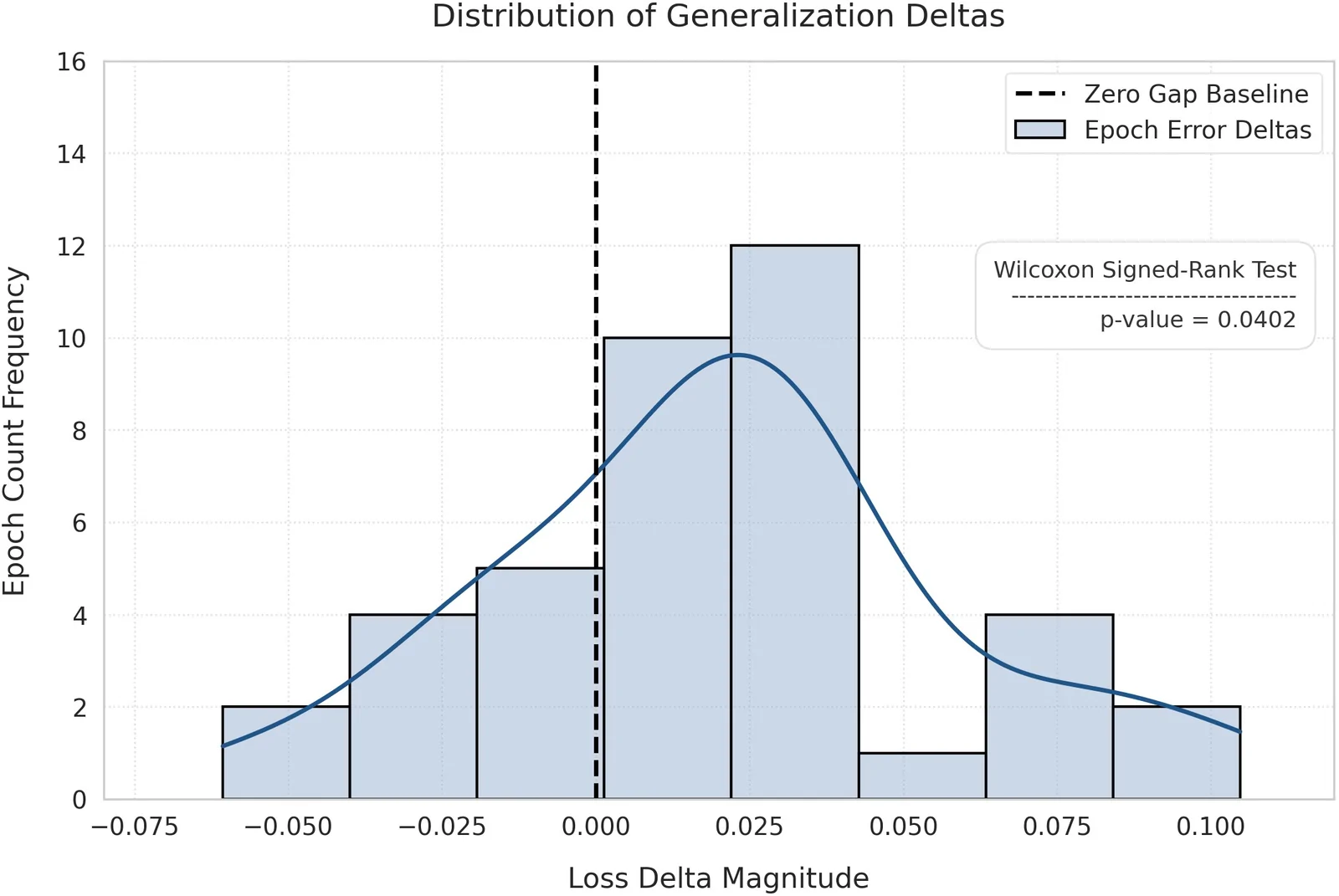
Distribution of epoch-wise error deltas, illustrating the strong training stability and consistent convergence of the proposed network.

Despite these strong generalization capabilities, it is important to acknowledge a few failure cases observed during evaluation. As illustrated in Figure 10, TexTNet occasionally produces fragmented boundaries or suboptimal segmentations in regions where atypical stromal morphology visually mimics malignant glandular structures. Because these ambiguous regions can disrupt the LBP texture descriptors, future work will explore integrating uncertainty-aware learning to better flag these specific areas for clinical review.

**Figure 10:**
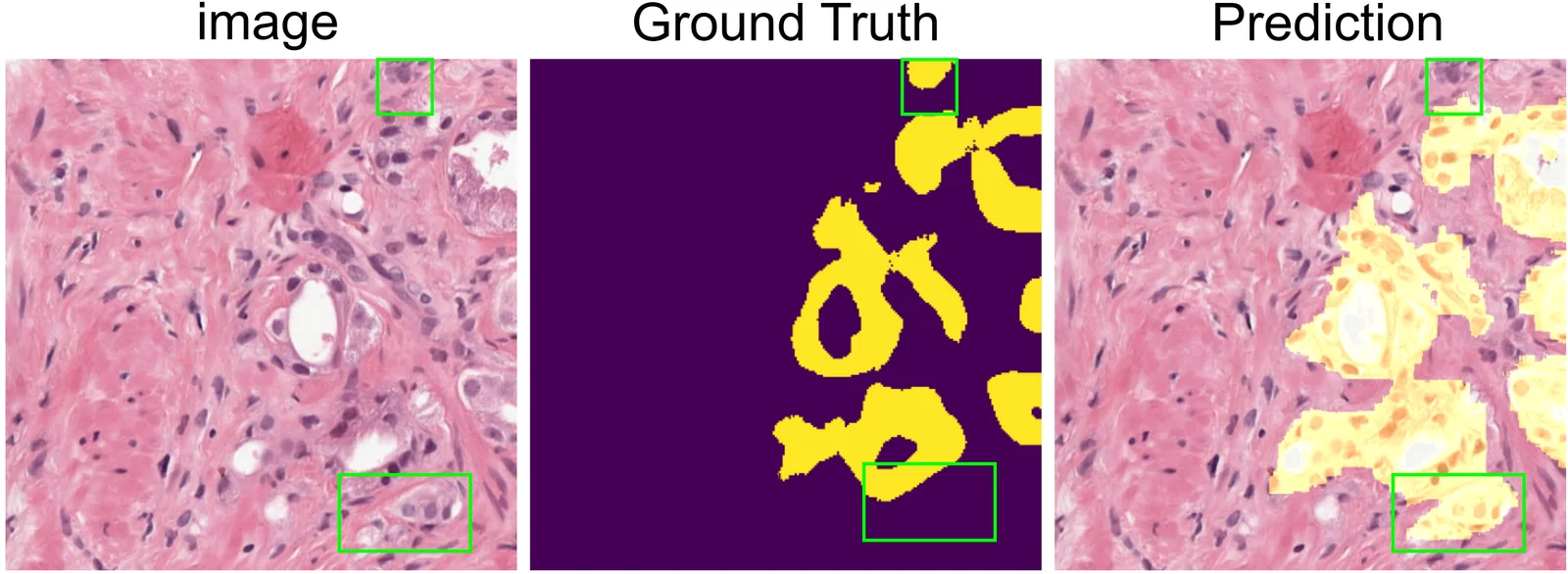
Failure case on PANDA dataset.

### 5.3 Discussion

In this study, we proposed TexTNet, a novel deep learning architecture for prostate cancer Gleason grade segmentation in WSIs. The integration of Swin Transformer for global context extraction and texture-sensitive attention from LBP features significantly improved segmentation performance. The combination of these two approaches allows TexTNet to effectively model both fine-grained tissue details and global context, which are crucial for accurate Gleason grading. Our results on the SICAPv2 and PANDA datasets demonstrate that TexTNet outperforms SOTA methods across multiple regions of interest, including high-resolution segments such as G3, G4, and G5 Gleason grades, as well as prostate zones like TZ and PZ. In particular, TexTNet achieved higher DSC scores for the G3, G4, and G5 regions compared to methods like TransUNet, MFTrans, DHUnet, CTNet, and MaskHIT, showcasing its ability to capture both subtle tissue variations and complex boundaries.

The ACB proved essential in enhancing local feature extraction, particularly in areas with dense tissue structures. By reinforcing both horizontal and vertical edge features, ACB refined the segmentation, especially in regions with intricate structures. The CDFA further contributed to TexTNet’s performance by aggregating global and local features, enhancing its ability to capture multi-scale information and suppress background noise. The RSegH ensured final feature aggregation, producing sharp, accurate segmentation maps across all regions, with a notable improvement in G5. The DP-SFE module, in particular, played a critical role in bridging the gap between global and local features. By recalibrating feature responses based on both global context and fine-grained details, DP-SFE mitigated boundary blurring, which is a common challenge in complex tissue segmentation tasks. This allowed TexTNet to achieve superior performance in areas where other methods struggled, particularly in delineating tissue boundaries.

Comparing TexTNet to the SOTA models, our method demonstrates the benefits of combining Swin Transformer’s long-range dependency modeling with the LBP-guided attention mechanism. While models like TransUNet and MFTrans perform well by incorporating Transformer-based architectures, they do not leverage the texture-sensitive attention provided by TexTNet, which enhances its capability to detect fine-grained tissue structures. Furthermore, TexTNet outperforms models like MaskHIT and CTNet, which rely on less refined feature fusion or pretraining strategies, by offering a more comprehensive approach that integrates local feature refinement with global context. In summary, TexTNet’s combination of Swin Transformer, LBP-guided attention, and advanced feature aggregation modules (ACB, CDFA, and RSegH) leads to significant improvements in segmentation performance for prostate cancer Gleason grading in WSIs. The results on the SICAPv2 and PANDA datasets, as well as the HuBMAP dataset, highlight the robustness and versatility of TexTNet in handling a variety of segmentation tasks, particularly for complex tissue regions. TexTNet demonstrates inherent cross-modality generalizability on the ProstateX dataset without explicit domain adaptation. Because the LBP descriptor relies on relative pixel inequalities rather than absolute intensities, it seamlessly translates from WSI stain gradients to MRI grayscale textures. Additionally, the DP-SFE module acts as an implicit domain adapter by dynamically recalibrating feature channels to isolate robust structural features across both domains. To rigorously evaluate robust generalization beyond primary H&E stained WSIs, we benchmarked TexTNet on the ProstateX and HuBMAP datasets. Evaluating on the MRI-based ProstateX dataset demonstrates cross-modality generalizability, proving that our attention layers seamlessly translate to grayscale radiology textures without structural alterations. Concurrently, utilizing the PAS-stained HuBMAP dataset validates cross-stain adaptability, establishing that TexTNet maintains precise segmentation across disparate laboratory staining protocols.

## 6 Conclusion

In this work, we presented TexTNet, a novel architecture for prostate cancer Gleason grade segmentation in WSIs. By combining the strengths of Swin Transformer for global feature extraction and LBP-guided attention for texture refinement, TexTNet effectively captures both global context and fine-grained tissue details. The integration of ACB, CDFA, and RSegH further enhances segmentation performance, making TexTNet the top-performing model across multiple datasets, including SICAPv2, PANDA, and HuBMAP. The results demonstrate significant improvements in DSC scores, particularly in challenging regions such as G3, G4, G5, and Urethra, when compared to existing state-of-the-art methods. The ability of TexTNet to model both global context and local texture-sensitive details makes it a powerful tool for accurate prostate cancer grading and other medical image segmentation tasks.

### 6.1 Limitation

A key limitation is that managing multi-scale complexity with advanced transformer architectures incurs significant computational overhead, hindering real-time deployment in resource-constrained clinical settings without specialized hardware.

### 6.2 Future work

Will focus on extending TexTNet to handle multimodal datasets, combining histopathology with radiology for improved generalization. Additionally, exploring self-supervised learning methods can help reduce dependency on annotated data. Optimizing the model for faster inference on larger WSIs and deploying lightweight versions for real-time applications will also be crucial for clinical implementation.

## Data Availability

Publicly available datasets were analyzed in this study. This data can be found here: https://data.mendeley.com/datasets/9xxm58dvs3/1 https://panda.grand-challenge.org/data/ https://www.kaggle.com/competitions/hubmap-organ-segmentation https://www.cancerimagingarchive.net/collection/prostatex/.
